# Quantitative X-ray pair distribution function analysis of nanocrystalline calcium silicate hydrates: a contribution to the understanding of cement chemistry

**DOI:** 10.1107/S1600576716017404

**Published:** 2017-02-01

**Authors:** Sylvain Grangeon, Alejandro Fernandez-Martinez, Alain Baronnet, Nicolas Marty, Agnieszka Poulain, Erik Elkaïm, Cédric Roosz, Stéphane Gaboreau, Pierre Henocq, Francis Claret

**Affiliations:** a D3E/SVP, BRGM (French Geological Survey), 3 Avenue Claude Guillemin, Orléans, 45060, France; b ISTerre, CNRS and Université Grenoble Alpes, Grenoble, 38041, France; c CINaM UMR 7325, Université Aix-Marseille, Marseille, 13288, France; d CINaM UMR 7325, CNRS, Marseille, 13288, France; e ESRF, The European Synchrotron, 71 Avenue des Martyrs, Grenoble, 38000, France; f Synchrotron Soleil, L’Orme des Merisiers Saint-Aubin, Gif-sur-Yvette Cedex, BP 48 91192, France; g Scientific Division, Andra, 1-7 Rue Jean Monnet, Parc de la Croix Blanche, Chatenay-Malabry Cedex, 92298, France

**Keywords:** C–S–H, calcium silicate hydrates, pair distribution function, transmission electron microscopy

## Abstract

The structural evolution of nanocrystalline calcium silicate hydrate (C–S–H) as a function of its calcium to silicon (Ca/Si) ratio has been probed using qualitative and quantitative X-ray atomic pair distribution function analysis of synchrotron X-ray scattering data. Whatever the Ca/Si ratio, the C–S–H structure is similar to that of tobermorite. When the Ca/Si ratio increases from ∼0.6 to ∼1.2, Si wollastonite-like chains progressively depolymerize through preferential omission of Si bridging tetrahedra. When the Ca/Si ratio approaches ∼1.5, nanosheets of portlandite are detected in samples aged for 1 d, while microcrystalline portlandite is detected in samples aged for 1 year. High-resolution transmission electron microscopy imaging shows that the tobermorite-like structure is maintained to Ca/Si > 3.

## Abstract 2

Quantitative analysis of the X-ray pair distribution function collected on calcium silicate hydrates having Ca/Si ratios ranging between 0.57 and 1.47 was applied. With increasing Ca/Si ratio, Si bridging tetrahedra are omitted and Ca(OH)_2_ is detected at the highest ratios.

## 1. Introduction   

Nanocrystalline calcium silicate hydrate (C–S–H) is the main hydration product of many types of cements (Richardson, 2008 ▸), including ordinary Portland cement (OPC). It controls the main mechanical (Manzano *et al.*, 2007 ▸) and chemical properties (Blanc *et al.*, 2010 ▸) of the hydrated cement-based materials. As more than 7 × 10^9^ m^3^ of OPC are produced each year (Richardson, 2014 ▸), C–S–H is probably the most abundant man-made material on Earth.

C–S–H has variable crystal chemistry, which can be expressed as Ca*_x_*Si*_y_*O_(*x*+2*y*)_·*n*H_2_O, where *y* typically ranges between 2 and 3, *x*/*y* between 0.6 and 2.2, and *n* depends on many parameters such as the abundance of interlayer Ca or the hydration state (Feldman & Sereda, 1970 ▸; Groves *et al.*, 1986 ▸; Nonat & Lecoq, 1996 ▸; Richardson, 2004 ▸, 2008 ▸; Taylor, 1997 ▸). At an *x*/*y* ratio (Ca/Si ratio) lower than ∼1.25, C–S–H is thought to resemble nanocrystalline and disordered tobermorite (Allen & Thomas, 2007 ▸; Allen *et al.*, 2007 ▸; Grangeon, Claret, Lerouge *et al.*, 2013 ▸; Nonat, 2004 ▸; Richardson, 2004 ▸; Skinner *et al.*, 2010 ▸; Vandamme & Ulm, 2013 ▸), a mineral built of layers of Ca atoms coordinated to seven O atoms sandwiched between chains of wollastonite-like Si tetrahedra. The structure of samples having a higher Ca/Si ratio has long been debated. Some studies have proposed a structure close to jennite (Gard & Taylor, 1976 ▸; Richardson, 2008 ▸; Taylor, 1986 ▸), another layered mineral, whereas others (Cong & Kirkpatrick, 1996 ▸; Grangeon, Claret, Linard *et al.*, 2013 ▸; Nonat, 2004 ▸; Richardson, 2014 ▸) proposed that it consists of a mix of defective tobermorite and calcium hydroxide [Ca(OH)_2_ – CH in the cement chemistry literature]. This latter hypothesis is favoured by some authors in view of the structural data (*e.g.* Cong & Kirkpatrick, 1996 ▸) and by the fact that the tobermorite-like model explains the capacity of C–S–H to take up many elements such as Al, K or Na (Bach *et al.*, 2013 ▸; Myers *et al.*, 2013 ▸, 2015 ▸). This model is also plausible from a thermodynamic point of view, as the chemical composition of water at equilibrium with C–S–H can be modelled using a combination of amorphous silica, a tobermorite-like component and CH (Walker *et al.*, 2007 ▸, 2016 ▸; Berner, 1992 ▸). According to nano-indentation studies, CH and tobermorite would be intimately mixed (with CH possibly filling the C–S–H interlayer space) at the lowest Ca/Si ratios where they coexist, and would form two separate discrete phases at higher ratios (Chen *et al.*, 2010 ▸). This is supported by powder X-ray diffraction, as portlandite (a crystalline form of CH) is regularly detected in C–S–H samples of Ca/Si ratio equal to at least ∼1.6 (Garbev, Beuchle *et al.*, 2008 ▸; Garbev, Bornefeld *et al.*, 2008 ▸; Renaudin *et al.*, 2009 ▸). However, despite abundant suggestions of the presence of CH in samples having a Ca/Si ratio in the range 1.25–1.5, direct evidence is still lacking.

In addition to the ambiguities concerning the potential presence of CH in samples having a Ca/Si ratio between ∼1.25 and ∼1.5, many other details of the C–S–H structure remain poorly understood. This includes the evolution of the C–S–H lattice parameters as a function of the Ca/Si ratio and the exact mechanisms of depolymerization of the Si chains. However, all this information is needed to better understand C–S–H reactivity, as it dictates the composition of equilibrium pore water (Walker *et al.*, 2007 ▸, 2016 ▸) and the mechanisms of trace element uptake (Schlegel *et al.*, 2004 ▸; Viallis *et al.*, 1999 ▸). The multiplicity of existing C–S–H structural models is at least partly due to the combination of C–S–H nanocrystallinity and of the extreme degree of structural disorder prevailing in its structure (*e.g.* Grangeon, Claret, Lerouge *et al.*, 2013 ▸; Grangeon, Claret, Linard *et al.*, 2013 ▸; Taylor, 1956 ▸; Cong & Kirkpatrick, 1996 ▸) which prevents the use of the usual X-ray diffraction pattern refinement algorithms. Many studies have circumvented this problem by using methods that probe the short- or medium-range order in the structure [*e.g.* NMR, Raman, synchrotron X-ray absorption, Fourier transformed infrared spectrometry (Cong & Kirkpatrick, 1996 ▸; Klur *et al.*, 1998 ▸; Lequeux *et al.*, 1999 ▸; Yu *et al.*, 1999 ▸)]. Although relevant and useful tools, these methods only provide a partial picture of the structure, and are by nature unable to provide a full structural model. Previous studies have demonstrated that the use of the pair distribution function (PDF) analysis of high-energy X-ray scattering data is a very efficient way to determine the short-range order of highly disordered phases (Billinge & Kanatzidis, 2004 ▸). In the case of C–S–H, this method has, up to now, only been qualitatively used for the study of C–S–H structure (Skinner *et al.*, 2010 ▸; Soyer-Uzun *et al.*, 2012 ▸) or carbonation mechanisms (Morandeau & White, 2015 ▸). We performed a quantitative PDF analysis of high-energy X-ray scattering data to study the evolution of C–S–H structure as a function of its Ca/Si ratio. Special attention was paid to determination of the evolution of Si chains and to identification of the possible CH in samples having a Ca/Si ratio ranging between ∼1.25 and ∼1.5.

## 2. Materials and methods   

### 2.1. Samples   

Two series of C–S–H samples were produced, with the aim of testing the influence of ageing on C–S–H structure. All syntheses were performed in a CO_2_-free glove box continuously flushed with N_2_. A first series of samples was synthesized by mixing appropriate amounts of Ca(OH)_2_ and amorphous SiO_2_ (Aerosil 200) in water to reach a given target Ca/Si ratio, and leaving the mixture to react for 1 d. The other series was synthesized by mixing CaO and amorphous SiO_2_ and allowing the reaction to run for 1 year. Once the desired ageing time was reached, samples were filtered, freeze-dried and left in closed containers in the glove box. Tobermorite was synthesized by mixing Ca(OH)_2_ and SiO_2_ in water and running the reaction for 8.5 h at 453 K and 1 MPa. In addition, commercial high-purity (>99.9%) portlandite and amorphous silica were purchased to serve as standards. Finally, to check if the samples synthesized using the method described above could be representative of hydrated cement pastes, a sample was created by hydrating synthetic alite (C_3_S) in the presence of additional CaCl_2_, so as to reach a Ca/Si ratio of ∼3.

### 2.2. Electron probe micro-analyser   

Electron probe micro-analyser (EPMA) analyses were performed using a Cameca SX50 electron microprobe (acceleration voltage of 15 kV, beam current of 12 nA) and a 1–2 µm beam width. Prior to analysis, a 10–20 nm-thick carbon layer was sputter-coated on the samples (Edwards Auto 306). Ca and Si were analysed simultaneously. Ca *K*α and Si *K*α were analysed using a pentaerythritol crystal and a thallium acid phthalate crystal, respectively. The standards used were albite (NaAlSi_3_O_8_) for Si and wollastonite (CaSiO_3_) for Ca. A ZAF data correction (Merlet, 1994 ▸) was applied to the raw data.

### 2.3. High-energy X-ray scattering   

Tobermorite and the first series of C–S–H samples (1 d ageing time) were measured in 1 mm polyimide capillaries at beamline ID15B at the ESRF (European Synchrotron Radiation Facility, Grenoble, France) using an energy of 87 keV (λ = 0.142 Å). Instrument calibration was done using a NIST-certified CeO_2_ standard. A PerkinElmer flat panel detector was used to detect X-ray scattering. Data were automatically corrected for internal dark current and the signal from the empty capillary was subtracted as a background. Forty frames were collected for each sample, with acquisition time ranging between 5 and 12 s, adjusted to optimize the signal-to-noise ratio, and the obtained data were integrated and averaged in *Fit2D* (Hammersley, 2016 ▸). The second series of samples (1 year ageing time) was measured at beamline CRISTAL at the SOLEIL synchrotron (French National Synchrotron Facility, Paris, France) using an energy of 28 keV (λ = 0.436 Å) and an XPad hybrid pixel detector. Data were collected in the 1.2–124.5° 2θ angular range with a total collection time of 30 min, owing to the fact that the detector had to record the data set step by step, and were processed with specific software (Ounsy *et al.*, 2013 ▸).

All diffraction data were Fourier transformed to PDF data using *PDFgetX3* (Juhás *et al.*, 2013 ▸) and data simulation was performed either using *PDFGui* (Farrow *et al.*, 2007 ▸), for all C–S–H and tobermorite PDF data, with tobermorite (Merlino *et al.*, 2001 ▸) as a structure model, or using the *Diffpy-CMI* framework (Gagné & Hawthorne, 2015 ▸), for analysis of differential PDF data. The full structure model used for PDF data modelling is available in the supporting information. Reciprocal-space broadening and damping factors were, respectively, refined to *Q*_broad_ = 0.048 Å^−1^ and *Q*_damp_ = 0.044 Å^−1^ for ESRF data and *Q*_broad_ = 0.02 Å^−1^ and *Q*_damp_ = 0.01 Å^−1^ for SOLEIL data. The upper *q* value used for the calculation of PDF data (*Q*_max_) was equal to 16.7 Å^−1^ for ESRF data and 12 Å^−1^ for SOLEIL data. All data having the same target Ca/Si ratio were refined simultaneously, using the same structure model.

As the C–S–H Ca/Si ratio increases, the depolymerization of Si wollastonite chains is thought to proceed *via* preferential omission of bridging Si tetrahedra (*e.g.* Cong & Kirkpatrick, 1996 ▸). This was taken into account during the refinement of each data set by performing four independent calculations in which the occupancy of Si bridging and paired tetrahedra was constrained differently. In two of the four simulations, only the occupancy of bridging Si (calculation 1) or paired Si (calculation 2) tetrahedra was refined, the other one being set to 1. In a third simulation (calculation 3), the occupancy of both sites was constrained to be equal, and in a fourth simulation (calculation 4) both occupancies were refined independently. Simulation quality was evaluated using the usual goodness-of-fit factor [*R*_wp_ factor (Egami & Billinge, 2012 ▸)]. When calculations 2, 3 and 4 could not evidence the presence of vacancies in the paired Si tetrahedron sites, calculation 1 was preferred. Note that, in such cases, the difference between the highest and lowest *R*_wp_ was less than 2%. Otherwise, the simulation having the lowest *R*_wp_ was preferred. In the simulation of PDF data from samples having a target Ca/Si ratio of 0.6 (see below), the occupancy of both Si sites was constrained to be 1 as all scenarios converged to defect-free Si chains. Note that uncertainties are only indicative, as the uncertainties on the measurement itself are unknown.

### 2.4. High-resolution transmission electron microscopy (HRTEM)   

HRTEM work was performed on a JEOL JEM 3010 high-resolution transmission electron microscope working under 300 kV acceleration voltage at the electron microscopy facilities at CINaM. 50 µm-thick slates of the hydrated C_3_S were drilled as discs of 3 mm in diameter that were glued onto a transmission electron microscope Cu grid and then argon-ion milled up to transparency for electrons with a double-gun precision ion polishing system from Gatan, and eventually C-coated for further electron conduction. The quick observation and image recording on film under moderate magnification (140 000×) were designed to prevent beam damage (Grangeon, Claret, Lerouge *et al.*, 2013 ▸).

## 3. Results and discussion   

### 3.1. Qualitative analysis of X-ray diffraction patterns   

**Figure 1:**
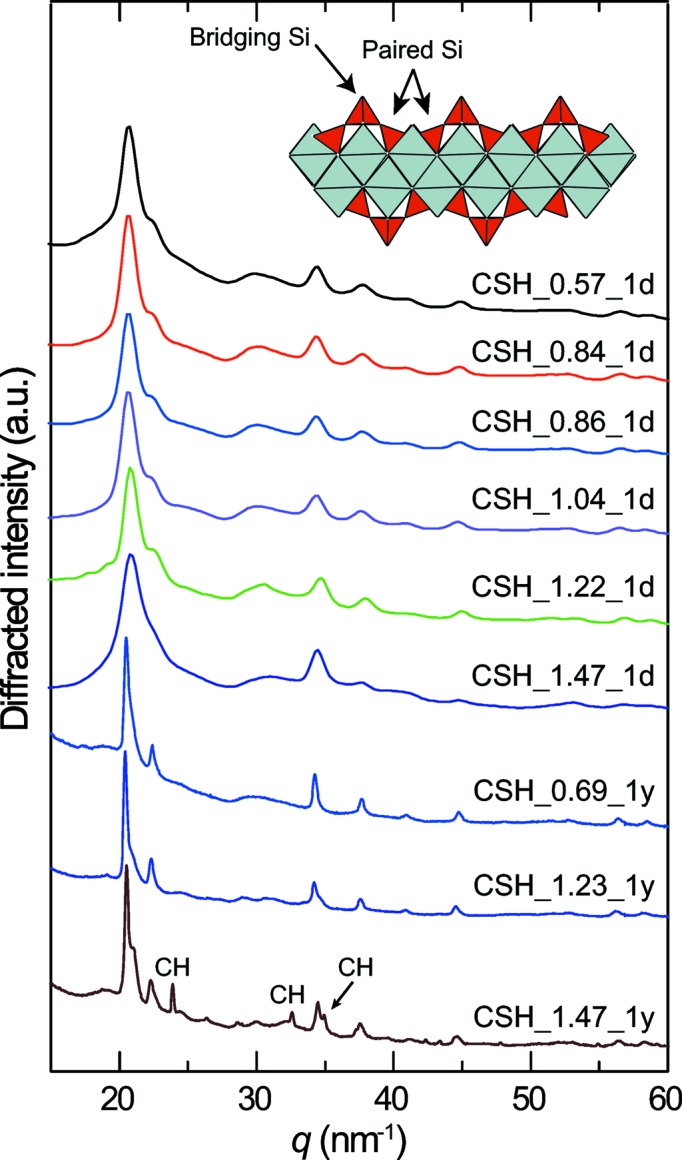
X-ray diffraction patterns of all C–S–H samples. From top to bottom, samples are sorted by increasing synthesis time and then by increasing Ca/Si ratio. The inset at the top right is a sketch of the C–S–H structure, with grey polyhedra representing (CaO_7_)^12−^ polyhedra and red tetrahedra representing (SiO_4_)^4−^ polyhedra. Two paired Si tetrahedra (two tetrahedra connected, at the surface of the Ca layer) and a bridging Si tetrahedron (bridging two of the aforementioned paired Si) are pointed out with arrows.

Sample labels, Ca/Si ratios and synthesis times are available in Table 1 ▸. Regardless of sample Ca/Si ratio and of synthesis time, all X-ray diffraction (XRD) patterns but that of CSH_1.47_1y are typical for C–S–H, with only a few maxima being expressed (Fig. 1 ▸), all of them being attributable to nanocrystalline and disordered tobermorite (Grangeon, Claret, Linard *et al.*, 2013 ▸). In the case of CSH_1.47_1y, additional maxima attributable to microcrystalline CH (portlandite) are present. The maxima are sharper but not more abundant in the patterns of samples left to age for 1 year compared with those left to age for 1 d. This shows that ageing time favoured crystal growth, but influenced neither layer structure nor stacking order. In contrast, the transformation of C–S–H to tobermorite was observed to occur within 8 d in an aqueous solution having a Ca/Si ratio of ∼0.8, heated at 423 K (Houston *et al.*, 2009 ▸). This indicates that the transformation of C–S–H to tobermorite may be triggered by temperature.

### 3.2. Qualitative and quantitative study of PDF data   

**Figure 2:**
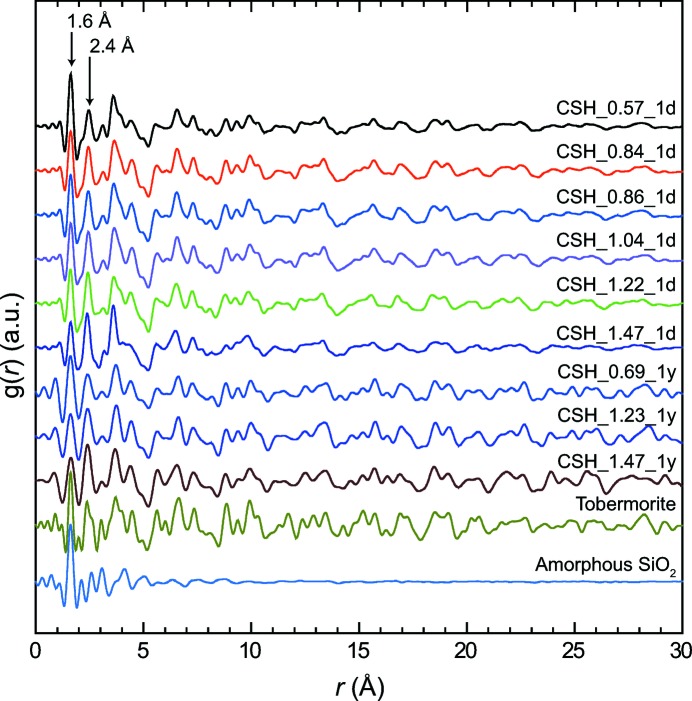
X-ray PDF of, from top to bottom, all C–S–H samples (sorted as in Fig. 1 ▸), tobermorite and amorphous SiO_2_.

**Figure 3:**
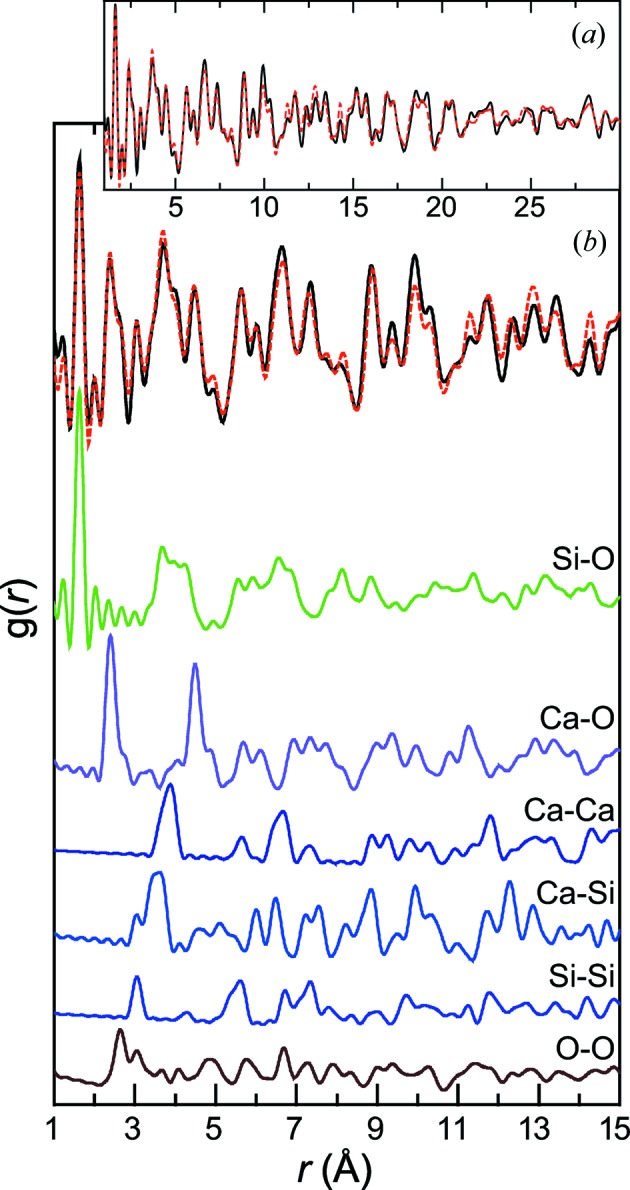
(*a*) Experimental (solid black line) and calculated (dashed red line) tobermorite PDF. (*b*) Focus on the 1–15 Å range of the experimental and calculated patterns (top) and, below, relative contributions of, from top to bottom, Si–O, Ca–O, Ca–Ca, Ca–Si, Si–Si and O–O atomic pairs to the total PDF.

Coherently with the XRD study, C–S–H PDF data are close to those of tobermorite (Fig. 2 ▸). For a given target Ca/Si ratio, the main difference between the PDF data of samples aged for 1 d and 1 year is the intensification of the structural features at greater interatomic distance *r* with time. For samples left in solution for 1 d, structural features disappear at *r* ≃ 20 Å, which demonstrates the strict nanocrystallinity of these compounds. Conversely, structural features are still observed at *r* = 20 Å and more in samples left to age for 1 year, meaning that they have a larger crystallite size. In both cases, the extent of the correlation proves that samples are not amorphous but rather hold long-range order. Indeed, if the samples were amorphous, then correlations would not extend to *r* values higher than a few ångströms, as exemplified with amorphous silica (Fig. 2 ▸). Further quantitative comparison of PDF data from the two series is prevented by the fact that they were not acquired with the same instrumental parameters and resolution. However, in each series, and as previously observed (Soyer-Uzun *et al.*, 2012 ▸), the intensity of the correlation at ∼1.6 Å decreases relative to that of the correlation at ∼2.4 Å when the sample Ca/Si ratio increases. As the integrated intensity of a PDF peak is directly related to the coordination number (Egami & Billinge, 2012 ▸), this implies an opposite trend in the relative abundances of two atomic pairs as a function of the Ca/Si ratio. To determine to which atomic pairs these correlations belong, the tobermorite data were fitted (see Table S1 and Fig. S1 in the supporting information for a full description of the structure model used), and the partial pair differential functions extracted (Fig. 3 ▸). From this simulation, the correlations at ∼1.6 and ∼2.4 Å can be attributed, respectively, to Si—O and Ca—O, and the relative evolution of their integrated intensity is thus a proxy for the relative abundance of Si and Ca atoms in the structure. It can therefore be concluded, in agreement with previous findings (Skinner *et al.*, 2010 ▸; Soyer-Uzun *et al.*, 2012 ▸), that the evolution of the Ca/Si ratio in a series of C–S–H samples can be deduced from the simple examination of these two correlations. This proves that PDF analysis is a valuable complementary method to ^29^Si NMR or XRD for C–S–H studies, for example for spatially resolved studies (*e.g.* analysis of a slice of cement-based material). At higher *r* values, all correlations have contributions of different atomic pairs and thus cannot be interpreted in a straightforward way.

**Figure 4:**
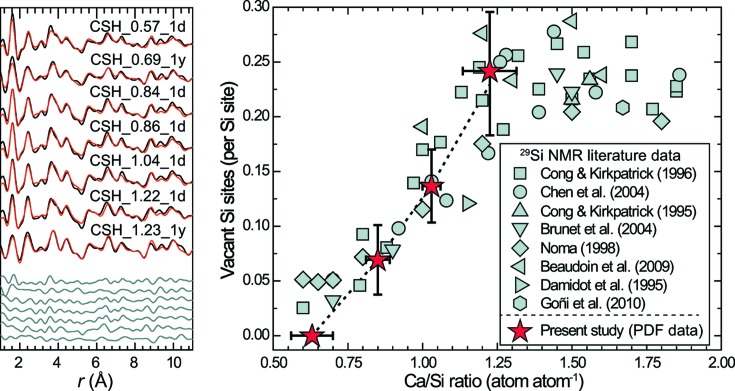
Left panel: experimental (black solid line), calculated (red solid line) and residual (grey solid line) PDFs of samples having target Ca/Si ratios of 0.6, 0.8, 1 and 1.2 sorted, from top to bottom, by increasing Ca/Si ratio and then by increasing synthesis time. Right panel: evolution of the abundance of Si vacancies as a function of sample Ca/Si ratio. The present data (filled red stars) are compared with literature data obtained using ^29^Si NMR [the symbols square, circle, triangles pointing up, down, left and right, diamond, and octahedron, respectively, refer to data from Cong & Kirkpatrick (1996 ▸), Chen *et al.* (2004 ▸), Cong & Kirkpatrick (1996 ▸), Brunet *et al.* (2004 ▸), Beaudoin *et al.* (2009 ▸), Damidot *et al.* (1995 ▸), Noma *et al.* (1998 ▸) and Goñi *et al.* (2010 ▸)].

To further characterize C–S–H structure evolution when the Ca/Si ratio increases, data were fitted using a tobermorite model and, by analogy to other turbostratic phases (Grangeon *et al.*, 2015 ▸; Manceau *et al.*, 2013 ▸), the fitting procedure was restricted to the 1–11 Å range to minimize the influence of stacking disorder. Refinement of the PDFs from CSH_1.47_1d and CSH_1.47_1y failed because these samples contain two phases, as discussed below. Consequently, only the PDF data of samples having a target Ca/Si ratio of 1.2 or less and their best simulation are shown in Fig. 4 ▸, and the main refined parameters are reported in Table 2 ▸. When the target Ca/Si ratio increases from 0.6 to 1, the number of vacancies in the Si chains increases from 0 to 0.14 ± 0.03, the bridging Si tetrahedra (inset in Fig. 1 ▸) being certainly preferentially omitted, although the four simulations (differing in the way Si vacancies are introduced – see §2.3) led to statistically identical results. A further increase of the Ca/Si ratio, up to 1.2, is accompanied by an increase in the number of Si vacancies, up to 0.24 ± 0.06, with Si bridging tetrahedra being preferentially removed (Table S2). As observed for other structures (Fernandez-Martinez *et al.*, 2010 ▸; Grangeon *et al.*, 2015 ▸; Manceau *et al.*, 2013 ▸), the sensitivity of PDF modelling to the composition of the interlayer was lower than for the composition of the layer. Because of this and of the lack of external constraints on the composition of the interlayer [*e.g.* abundance of interlayer water which depends on many factors such as relative humidity (Roosz *et al.*, 2016 ▸)], the structure of the interlayer was not refined. In particular, no attempt was made to determine if part of the interlayer water could in fact be interlayer Ca, which is certainly present at Ca/Si ≥ ∼5/6 (Cong & Kirkpatrick, 1996 ▸; Grangeon, Claret, Lerouge *et al.*, 2013 ▸; Richardson, 2008 ▸, 2014 ▸).

### 3.3. Comparison with ^29^Si NMR data   

Results from PDF data simulation compare well with ^29^Si NMR literature data (Beaudoin *et al.*, 2009 ▸; Brunet *et al.*, 2004 ▸; Chen *et al.*, 2004 ▸; Cong & Kirkpatrick, 1995 ▸, 1996 ▸; Damidot *et al.*, 1995 ▸; Goñi *et al.*, 2010 ▸; Noma *et al.*, 1998 ▸) (Fig. 4 ▸). A minor difference is observed in samples having a target Ca/Si of 0.6 which have defect-free Si chains according to the PDF analysis but contain ∼0.05 vacancy per Si site according to ^29^Si NMR. However, the number of Si vacancies estimated from ^29^Si NMR data is influenced by the presence of Si atoms at the crystal edge, and an accurate determination of the number of Si vacancies requires knowledge of the size of the crystal. In contrast, the PDF has no such dependence on border effects and, in addition, compared with ^29^Si NMR, it can probe the whole structure, including the Si site subjected to depolymerization, the atomic positions within the lattice and lattice parameters. Conversely, ^29^Si NMR provides direct insight into the local environment of Si regardless of the number and nature of the different phases building up the samples, showing that, when the Ca/Si ratio is ∼1.3 or higher, the Si abundance in the wollastonite-like chains is constant (Fig. 4 ▸). This implies that the increase in the Ca/Si ratio above ∼1.3 is solely due to the incorporation of Ca, either in the C–S–H structure or (and) as a discrete phase. It was demonstrated above that CSH_1.47_1y contains microcrystalline CH (Fig. 1 ▸), which affects its bulk Ca/Si ratio and explains why its PDF cannot be reproduced with a tobermorite-like model. We wonder if this is also the case for CSH_1.47_1d, although its XRD pattern does not evidence the presence of such a phase.

### 3.4. Evidence for the presence of Ca(OH)_2_ nanosheets in samples of high Ca/Si ratio   

**Figure 5:**
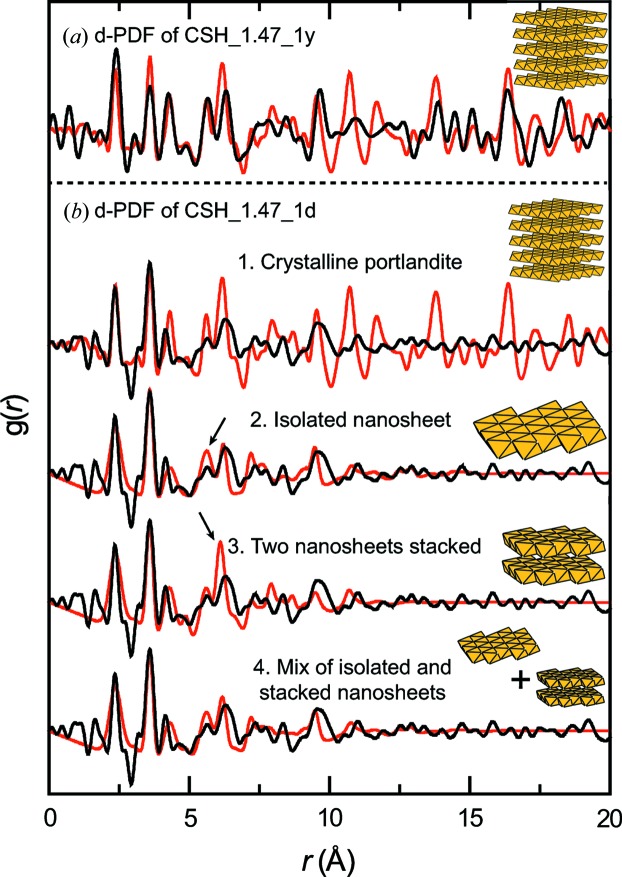
Differential PDF data obtained from CSH_1.47_1d and CSH_1.47_1y. Top panel (*a*) compares the d-PDF from CSH_1.47_1y (solid black line) and the experimental PDF of a portlandite standard (solid red line). A scheme of the portlandite structure is shown in the top right corner (yellow octahedra represent the O atoms which form the first shell around Ca). Bottom panel (*b*) compares the d-PDF from CSH_1.47_1d, from top to bottom, with the same experimental portlandite data as in panel (*a*) (sub-panel 1: structure model shown on the right side), with the calculated PDF of a portlandite-like nanosheet (sub-panel 2: structure model shown on the right side shows the full structure used for calculation; this model contains 17 Ca atoms coordinated to six O, forming regular octahedra, with Ca—O distances = 2.366 Å and Ca—Ca distances = 3.586 Å), with the calculated PDF of a stack of two of the portlandite-like nanosheets (sub-panel 3: structure model shows the full structure used for calculation), and with the calculated PDF of a 2:1 mix of portlandite nanosheets, identical to those of panel 2, and of stacks of two nanosheets, identical to those of sub-panel 3 (sub-panel 4). Note that the portlandite nanosheet has many similarities with the interlayer Ca(OH)_2_ proposed by several previous studies (Garbev, Bornefeld *et al.*, 2008 ▸; Grangeon *et al.*, 2016 ▸; Richardson, 2014 ▸).

To determine if CSH_1.47_1d contains CH, the differential PDF (d-PDF) method was employed. CSH_1.47_1y was also studied with d-PDF to check if this method is efficient in determining the presence of CH in a C–S–H sample. d-PDF data were obtained by subtracting the scaled PDF of CSH_1.22_1d and CSH_1.23_1y from those of CSH_1.47_1d and CSH_1.47_1y (Fig. 5 ▸), with the aim of isolating a contribution attributable to a Ca-rich phase. Despite potential minor inaccuracies (*e.g.* C–S–H lattice parameters in CSH_1.22_1d and CSH_1.23_1y on the one hand and CSH_1.47_1d and CSH_1.47_1y on the other may differ slightly), the two d-PDFs reveal several correlations, meaning that CSH_1.47_1d and CSH_1.47_1y contain a second phase in addition to the tobermorite-like phase. As the two d-PDFs have similar correlations up to ∼10 Å, it can be safely assumed that this second phase is the same in the two samples, the main difference being a larger crystallite size in CSH_1.47_1y, because its d-PDF has correlations up to *r* > 20 Å.

Comparison of the d-PDF from CSH_1.47_1y with the PDF of the portlandite standard leads to a satisfying agreement, which validates the use of d-PDF to identify the presence of CH in a C–S–H sample. Some correlations found in the portlandite PDF between 8 and 12 Å are absent in the d-PDF, which might be due to stacking disorder in the portlandite crystals from CSH_1.47_1y. In contrast, the d-PDF of CSH_1.47_1d is in poor agreement with the reference portlandite PDF, especially at *r* > 10 Å, where the experimental PDF vanishes (Fig. 5 ▸ *b*.1). A better agreement is obtained when data are compared with a calculated PDF of a single nanosheet of portlandite (Fig. 5 ▸ *b*.2), but a minor misfit is observed in the ratio of intensities at 5.6 and 6.3 Å, which is overestimated in the model. When the PDF of a crystal built of a stack of two of these nanosheets is calculated (Fig. 5 ▸ *b*.3), the ratio of the intensity of these two correlations is, oppositely, underestimated. The best agreement with data is obtained by assuming that the d-PDF signal is due to a mixture of isolated nanosheets and of crystallites built of two nanosheets stacked, with a relative ratio of 2:1 (Fig. 5 ▸ *b*.4). Thus, the main difference between CSH_1.47_1d and CSH_1.47_1y lies in the size of the portlandite crystallites: they are nanometric in the former and micrometric in the latter. Such a difference may be indicative of a kinetically controlled portlandite crystal growth mechanism, and could be understood as a coalescence phenomenon, although this might also result from the slightly different synthesis protocols used.

## 4. Relevance of present observations for the understanding of C–S–H formation in cement pastes   

**Figure 6:**
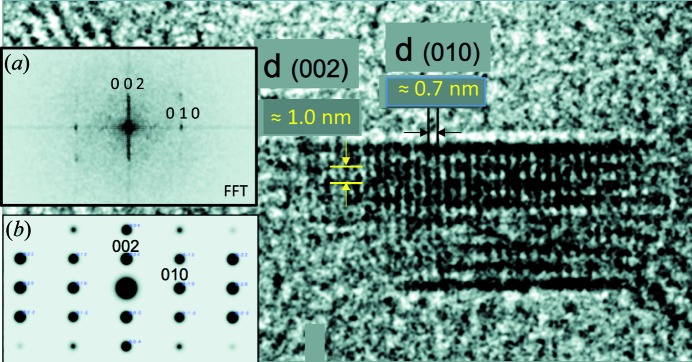
Main panel: HRTEM observation of a C–S–H crystal having a size of ∼6 × 20 nm, formed from hydration of C_3_S in the presence of additional CaCl_2_. Vertical and horizontal solid lines, respectively, highlight the presence of periodicities of ∼7 Å and, perpendicular, of 10–11 Å, which are more easily identified in the figure resulting from the fast Fourier transform of this image [inset (*a*)], this diagram being compatible with the calculated selected-area electron diffraction pattern [inset (*b*)] of tobermorite (Merlino *et al.*, 1999 ▸). Indexings are performed using the tobermorite (Merlino *et al.*, 1999 ▸) model.

While all present results provide a consistent picture of C–S–H structure evolution as a function of its Ca/Si ratio, we may wonder if they are applicable to real cement pastes, where C–S–H forms mainly from C_3_S hydration. In particular, it has been suggested that a jennite-like component could form at a high initial Ca/Si ratio (Richardson, 2008 ▸; Taylor, 1986 ▸). To test this hypothesis under extreme Ca/Si ratio conditions, the hydration of C_3_S in the presence of CaCl_2_, added so as to reach a very high Ca/Si ratio of ∼3, was monitored using HRTEM. As exemplified in Fig. 6 ▸, the lattice images of properly oriented particles formed in these conditions agree with lattice node projections of 11 Å tobermorite structure (Merlino *et al.*, 1999 ▸), with a layer-to-layer distance of 10–11 Å and a periodicity perpendicular to it nearly equal to 7 Å, compatible with the tobermorite *b* parameter (7.4 Å) but incompatible with the projection of the jennite lattice (Bonaccorsi *et al.*, 2004 ▸) along any direction within the layer. In addition, **b** is observed to be perpendicular to the direction of layer stacking, which is further evidence for the similarity between C–S–H and tobermorite and for the incompatibility between C–S–H and the triclinic jennite lattice. Interestingly, stacking disorder along the **b** lattice vector appears to be of limited amplitude. This means that C–S–H stacking disorder certainly mainly results from random translations along the **a** lattice vector.

Further comparison between this HRTEM image and results from PDF analysis, and in particular the potential presence of nanocrystalline CH intermixed with tobermorite-like layers, could not yet be performed for several reasons. The main reasons are that the actual Ca/Si ratio of this crystal could not be recorded owing to both its minute size and its limited stability under the beam. This image, however, is a significant step towards a better understanding of C–S–H structure, as it supports the absence of a jennite-like component at a high Ca/Si ratio.

## 5. Conclusion   

Coherently with previous literature studies, C–S–H was found to have a tobermorite-like structure, even at a high bulk Ca/Si ratio, as shown here with HRTEM. This supports the absence of a jennite-like component.

PDF analysis was shown to be a promising complementary method to ^29^Si NMR for the determination of the number of vacancies in the Si chains. Our study supports the idea that, when the C–S–H Ca/Si ratio increases from ∼0.6 to ∼1.2, the C–S–H structure evolution proceeds *via* depolymerization of the Si wollastonite-like chains, mainly *via* omission of Si bridging tetrahedra. This creates a layer charge deficit certainly compensated for by the incorporation of interlayer Ca, although this could not be quantitatively assessed in the present study. In samples with a higher Ca/Si ratio, portlandite was identified by using the differential PDF method.

All information gathered here supports the T/CH structure model for C–S–H (Richardson, 2014 ▸). Further developments could consist of applying the same methodology to the study of C–S–H samples saturated with water and (or) with aluminium incorporated, to contribute to a better understanding of C–S–H formation in cements (Richardson, 2014 ▸).

## Supplementary Material

Typical input file used for PDF data modelling in PDFGui. DOI: 10.1107/S1600576716017404/po5083sup1.pdf

## Figures and Tables

**Table 1:** Target and actual Ca/Si ratio of the C–S–H samples

Sample	Target Ca/Si (atom atom^−1^)	Synthesis time	Measured Ca/Si (atom atom^−1^)
CSH_0.57_1d	0.6	1 d	0.57 (5)
CSH_0.69_1y	0.6	1 year	0.69 (2)
CSH_0.84_1d	0.8	1 d	0.84 (3)
CSH_0.86_1d	0.8	1 d	0.86 (1)
CSH_1.04_1d	1.0	1 d	1.04 (3)
CSH_1.22_1d	1.2	1 d	1.22 (5)
CSH_1.23_1y	1.2	1 year	1.23 (4)
CSH_1.47_1d	1.5	1 d	1.47 (4)
CSH_1.47_1y	1.5	1 year	1.47 (5)

**Table 2:** Main structural parameters retrieved from modelling of C–S–H PDF data (all other parameters are available in the supporting information)

		Si occupancy			
Target Ca/Si	*a* (Å)†	Paired	Bridging	Bridging Si position along c* (Å)‡	*R*_wp_ (%)
0.6	6.6950 (100)	1	1	4.04 (3)	32.8
0.8	6.6838 (78)	1	0.79 (9)	4.16 (4)	31.6
1.0	6.7037 (110)	1	0.57 (13)	4.23 (9)	32.8
1.2	6.6825 (80)	0.91 (6)	0.45 (11)	4.14 (8)	32.4

Note: the uncertainty of the last digit is given as a number in brackets. Occupancies of 1 were fixed during the fitting procedure. For samples having a target Ca/Si ratio of 1.2, the simulation with the occupancy of paired tetrahedra set to 1 led to comparable *R*_wp_ (Table S2).

† *b* was constrained to be equal to 1.09651*a*. c* was not refined.

‡ Relative to the mean layer Ca plane.
