# Supplementary material for: Quantitative X-ray pair distribution function analysis of nanocrystalline calcium silicate hydrates: a contribution to the understanding of cement chemistry
Source: J Appl Crystallogr. 2017 Feb 1;50(Pt 1):14–21. doi: 10.1107/S1600576716017404 (PMC5294392; doi:10.1107/S1600576716017404)
Supplement: Supplementary file 1 [file j-50-00014-sup1.pdf]

JOURNAL OF APPLIED CRYSTALLOGRAPHY

Volume 49 (2016)

Supporting information for article:

Quantitative X-ray pair distribution function analysis of nanocrystalline calcium silicate hydrates: a contribution to the understanding of cement chemistry

Sylvain Grangeon, Alejandro Fernandez-Martinez, Alain Baronnet, Nicolas Marty, Agnieszka Poulain, Erik Elkaïm, Cédric Roosz, Stéphane Gaboreau, Pierre Henocq and Francis Claret

Table S1 All parameters refined during PDF data simulation but those reported in the main text. The corresponding input file is given in Figure S1.

Target Ca/Si ratio	Scale factor	Delta2 (Å^2)	Spdiameter (Å)	Ca and Si atoms		O atoms	
				u_{11} and u_{22} (Å^2)	u_{33} (Å^2)	u_{11} and u_{22} (Å^2)	u_{33} (Å^2)
0.6	1.08	2.52	28	0.010	0.002	0.004	0.04
0.8	1.17	2.54	37	0.006	0.008	0.007	0.07
1	1.17	2.52	41	0.006	0.009	0.007	0.06
1.2	1.18	2.52	30	0.005	0.008	0.008	0.06

Notes: delta2 is a quadratic atomic correlation factor. U_{33} associated to layer oxygen atoms are higher than the corresponding U_{11} and U_{22} values, certainly because the **c** lattice parameter was fixed during the modelling procedure so as to reduce the number of free parameters.

Table S2 Detail of the four PDF simulations performed on C-S-H samples having a target Ca/Si of 1.2.

Parameter	Constraints on occupancy of Si sites			
	Bridging sites refined	Paired sites refined	Equal occupancy of bridging and paired sites	Bridging and paired sites refined independently
a (Å)	6.685(8)	6.678(8)	6.680(8)	6.683(8)
Scale factor	1.16(6)	1.25(7)	1.23(7)	1.18(7)
Delta2 ($Å^2$)	2.5(1)	2.5(1)	2.5(1)	2.5(1)
Spdiameter (Å)	29(4)	31(4)	31(4)	30(4)
z-coordinate of bridging Si[§]	0.065(4)	0.067(1)	0.0678(2)	0.066(4)
Ca and Si U_{11} and U_{22} ($Å^2$)	0.005(1)	0.006(1)	0.005(1)	0.005(1)
Ca and Si U_{33} ($Å^2$)	0.007(3)	0.011(4)	0.011(4)	0.008(3)
O U_{11} and U_{22} ($Å^2$)	0.007(5)	0.007(2)	0.008(2)	0.008(2)
O U_{33} ($Å^2$)	0.06(1)	0.06(1)	0.06(1)	0.06(1)
Occupancy of bridging Si	0.37(9)	1[*]	0.78(4)[$]	0.45(11)
Occupancy of paired Si	1[*]	0.75(5)	0.78(4)[$]	0.91(6)
R_{WP}	32.70%	34.66%	35.74%	32.41%

Notes: [§]Relative to the model given in Figure S1; [*]fixed; [$]covaried

Figure S1: Typical PdfGUI input file used to model PDF patterns. Greyed parameters are those which were possibly refined (see main text and Table SI1).

Phase Configuration

a	6.73907	b	7.35343	c	22.487
alpha	90.0	beta	90.0	gamma	123.25

Scale Factor	0.972346				
delta1	0.0	delta2	2.57498	spdiameter	0.0
sratio	1.0	rcut	0.0	stepcut	0.0

Included Pairs: all-all

	elem	x	y	z	u11	u22	u33	u12	u13	u23	occ
1	Si	0.9087	0.7531	0.0712	0.0026164	0.0026164	0.00799335	0.0	0.0	0.0	1.0
2	Si	0.9087	0.7531	0.9288	0.0026164	0.0026164	0.00799335	0.0	0.0	0.0	1.0
3	Si	0.4087	0.7531	0.5712	0.0026164	0.0026164	0.00799335	0.0	0.0	0.0	1.0
4	Si	0.4087	0.7531	0.4288	0.0026164	0.0026164	0.00799335	0.0	0.0	0.0	1.0
5	O	0.771	0.5059	0.0942	0.00461025	0.00461025	0.0429663	0.0	0.0	0.0	1.0
6	O	0.771	0.5059	0.9058	0.00461025	0.00461025	0.0429663	0.0	0.0	0.0	1.0
7	O	0.271	0.5059	0.5942	0.00461025	0.00461025	0.0429663	0.0	0.0	0.0	1.0
8	O	0.271	0.5059	0.4058	0.00461025	0.00461025	0.0429663	0.0	0.0	0.0	1.0
9	O	0.759	0.178	0.1327	0.00461025	0.00461025	0.0429663	0.0	0.0	0.0	1.0
10	O	0.759	0.178	0.8673	0.00461025	0.00461025	0.0429663	0.0	0.0	0.0	1.0
11	O	0.259	0.178	0.6327	0.00461025	0.00461025	0.0429663	0.0	0.0	0.0	1.0
12	O	0.259	0.178	0.3673	0.00461025	0.00461025	0.0429663	0.0	0.0	0.0	1.0
13	O	0.985	0.5369	0.1982	0.00461025	0.00461025	0.0429663	0.0	0.0	0.0	1.0
14	O	0.985	0.5369	0.8018	0.00461025	0.00461025	0.0429663	0.0	0.0	0.0	1.0
15	O	0.485	0.5369	0.6982	0.00461025	0.00461025	0.0429663	0.0	0.0	0.0	1.0
16	O	0.485	0.5369	0.3018	0.00461025	0.00461025	0.0429663	0.0	0.0	0.0	1.0
17	O	0.519	0.3063	0.1942	0.00461025	0.00461025	0.0429663	0.0	0.0	0.0	1.0
18	O	0.519	0.3063	0.8058	0.00461025	0.00461025	0.0429663	0.0	0.0	0.0	1.0
19	O	0.019	0.3063	0.6942	0.00461025	0.00461025	0.0429663	0.0	0.0	0.0	1.0
20	O	0.019	0.3063	0.3058	0.00461025	0.00461025	0.0429663	0.0	0.0	0.0	1.0
21	O	0.894	0.746	0.0	0.00461025	0.00461025	0.0429663	0.0	0.0	0.0	1.0
22	O	0.394	0.746	0.5	0.00461025	0.00461025	0.0429663	0.0	0.0	0.0	1.0
23	O	0.188	0.893	0.094	0.00461025	0.00461025	0.0429663	0.0	0.0	0.0	1.0
24	O	0.188	0.893	0.906	0.00461025	0.00461025	0.0429663	0.0	0.0	0.0	1.0
25	O	0.688	0.893	0.594	0.00461025	0.00461025	0.0429663	0.0	0.0	0.0	1.0
26	O	0.688	0.893	0.406	0.00461025	0.00461025	0.0429663	0.0	0.0	0.0	1.0
27	O	0.27	0.434	0.0939	0.00461025	0.00461025	0.0429663	0.0	0.0	0.0	1.0
28	O	0.27	0.434	0.9061	0.00461025	0.00461025	0.0429663	0.0	0.0	0.0	1.0
29	O	0.77	0.434	0.5939	0.00461025	0.00461025	0.0429663	0.0	0.0	0.0	1.0
30	O	0.77	0.434	0.4061	0.00461025	0.00461025	0.0429663	0.0	0.0	0.0	1.0
31	O	0.77	0.86	0.0951	0.00461025	0.00461025	0.0429663	0.0	0.0	0.0	1.0
32	O	0.77	0.86	0.9049	0.00461025	0.00461025	0.0429663	0.0	0.0	0.0	1.0
33	O	0.27	0.86	0.5951	0.00461025	0.00461025	0.0429663	0.0	0.0	0.0	1.0
34	O	0.27	0.86	0.4049	0.00461025	0.00461025	0.0429663	0.0	0.0	0.0	1.0
35	O	0.523	0.811	0.1951	0.00461025	0.00461025	0.0429663	0.0	0.0	0.0	1.0
36	O	0.523	0.811	0.8049	0.00461025	0.00461025	0.0429663	0.0	0.0	0.0	1.0
37	O	0.023	0.811	0.6951	0.00461025	0.00461025	0.0429663	0.0	0.0	0.0	1.0
38	O	0.023	0.811	0.3049	0.00461025	0.00461025	0.0429663	0.0	0.0	0.0	1.0
39	O	0.987	0.0459	0.1985	0.00461025	0.00461025	0.0429663	0.0	0.0	0.0	1.0
40	O	0.987	0.0459	0.8015	0.00461025	0.00461025	0.0429663	0.0	0.0	0.0	1.0
41	O	0.487	0.0459	0.6985	0.00461025	0.00461025	0.0429663	0.0	0.0	0.0	1.0
42	O	0.487	0.0459	0.3015	0.00461025	0.00461025	0.0429663	0.0	0.0	0.0	1.0
43	Si	0.7581	0.3862	0.1574	0.0026164	0.0026164	0.00799335	0.0	0.0	0.0	1.0
44	Si	0.7581	0.3862	0.8426	0.0026164	0.0026164	0.00799335	0.0	0.0	0.0	1.0
45	Si	0.2581	0.3862	0.6574	0.0026164	0.0026164	0.00799335	0.0	0.0	0.0	1.0
46	Si	0.2581	0.3862	0.3426	0.0026164	0.0026164	0.00799335	0.0	0.0	0.0	1.0
47	Si	0.7592	0.9697	0.1577	0.0026164	0.0026164	0.00799335	0.0	0.0	0.0	1.0
48	Si	0.7592	0.9697	0.8423	0.0026164	0.0026164	0.00799335	0.0	0.0	0.0	1.0
49	Si	0.2592	0.9697	0.6577	0.0026164	0.0026164	0.00799335	0.0	0.0	0.0	1.0
50	Si	0.2592	0.9697	0.3423	0.0026164	0.0026164	0.00799335	0.0	0.0	0.0	1.0
51	Ca	0.2651	0.4328	0.2056	0.0026164	0.0026164	0.00799335	0.0	0.0	0.0	1.0
52	Ca	0.2651	0.4328	0.7944	0.0026164	0.0026164	0.00799335	0.0	0.0	0.0	1.0
53	Ca	0.7651	0.4328	0.7056	0.0026164	0.0026164	0.00799335	0.0	0.0	0.0	1.0
54	Ca	0.7651	0.4328	0.2944	0.0026164	0.0026164	0.00799335	0.0	0.0	0.0	1.0
55	Ca	0.7499	0.9228	0.2935	0.0026164	0.0026164	0.00799335	0.0	0.0	0.0	1.0
56	Ca	0.7499	0.9228	0.7065	0.0026164	0.0026164	0.00799335	0.0	0.0	0.0	1.0
57	Ca	0.2499	0.9228	0.7935	0.0026164	0.0026164	0.00799335	0.0	0.0	0.0	1.0
58	Ca	0.2499	0.9228	0.2065	0.0026164	0.0026164	0.00799335	0.0	0.0	0.0	1.0
59	O	0.427	0.219	0.0	0.0524101	0.0524101	0.228507	0.0	0.0	0.0	0.25
60	O	0.927	0.219	0.5	0.0524101	0.0524101	0.228507	0.0	0.0	0.0	0.25
61	O	0.879	0.237	0.0	0.0524101	0.0524101	0.228507	0.0	0.0	0.0	0.25
62	O	0.379	0.237	0.5	0.0524101	0.0524101	0.228507	0.0	0.0	0.0	0.25
63	O	0.422	0.8	0.0	0.0524101	0.0524101	0.228507	0.0	0.0	0.0	0.25
64	O	0.922	0.8	0.5	0.0524101	0.0524101	0.228507	0.0	0.0	0.0	0.25
